# Supplementary material for: Contact lenses, the reverse Pulfrich effect, and anti-Pulfrich monovision corrections
Source: Sci Rep. 2020 Sep 30;10:16086. doi: 10.1038/s41598-020-71395-y (PMC7527565; doi:10.1038/s41598-020-71395-y)
Supplement: Supplementary file 1 — Supplementary Figures. [file 41598_2020_71395_MOESM1_ESM.pdf]

Supplement:
Contact lenses, the reverse Pulfrich effect, and anti-Pulfrich monovision corrections

Victor Rodriguez-Lopez[1,2], Carlos Dorronsoro[1,3,†], Johannes Burge[2,4,5,†,*]

[1] Institute of Optics, Spanish National Research Council (IO-CSIC), Madrid, Spain
[2] Department of Psychology, University of Pennsylvania, Pennsylvania PA
[3] 2Eyes Vision SL, Madrid, Spain
[4] Neuroscience Graduate Group, University of Pennsylvania, Pennsylvania PA
[5] Bioengineering Graduate Group, University of Pennsylvania, Pennsylvania PA
[*] Correspondence to jburge@psych.upenn.edu
[†] Joint last-authorship

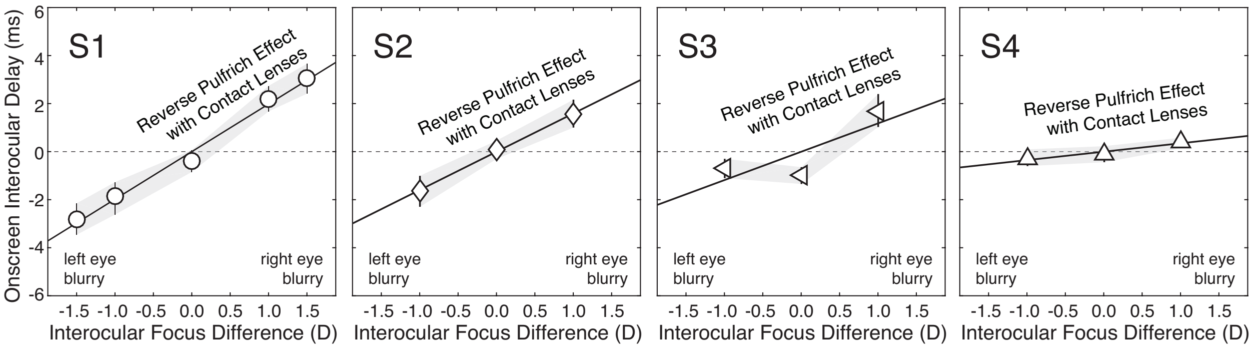

Figure S1. Reverse Pulfrich effect with contact lenses for all four human observers (Exp. 1). Onscreen interocular delays required to null neural differences in processing speed that are induced by differences in optical power between the eyes. Error bars indicate 68% confidence intervals on each PSE (i.e. point of subjective equality) from 1000 bootstrapped datasets.

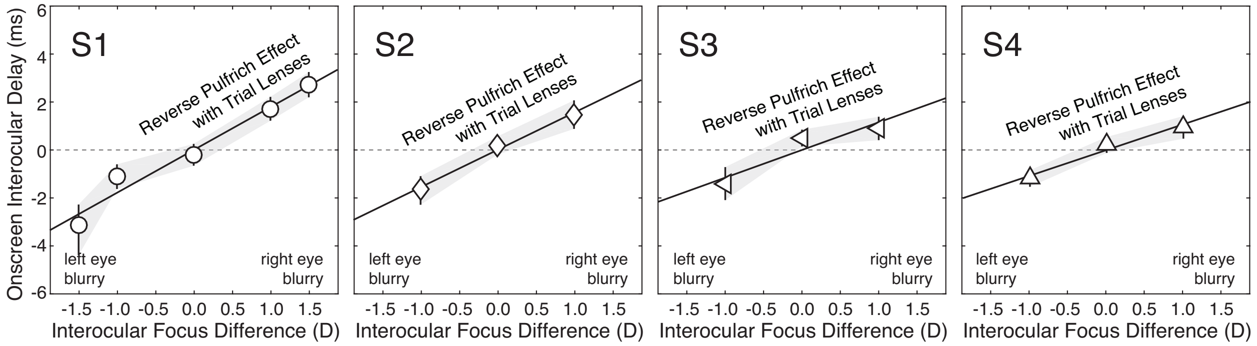

Figure S2. Reverse Pulfrich effect with trial lenses for all four human observers (Exp. 2). Onscreen interocular delays required to null neural differences in processing speed that are induced by differences in optical power between the eyes. Error bars indicate 68% confidence intervals on each PSE (i.e. point of subjective equality) from 1000 bootstrapped datasets.

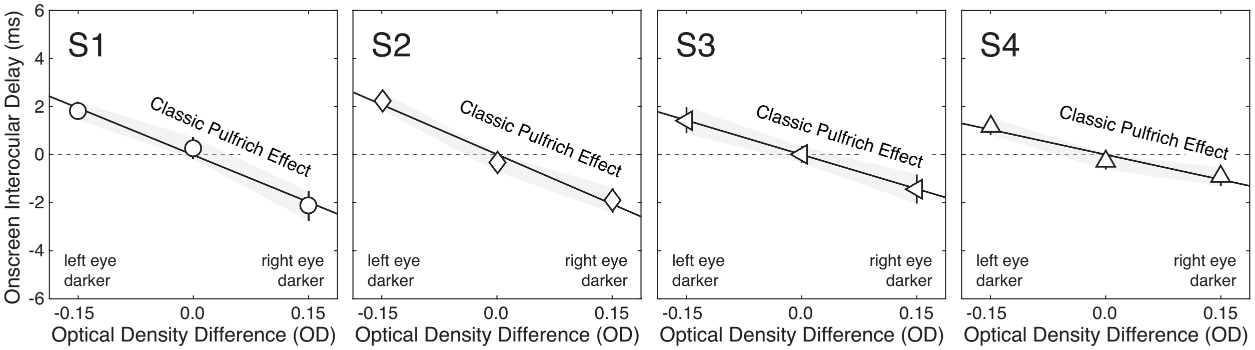

Figure S3. Classic Pulfrich effect with luminance differences for all four human observers (Exp. 3). Onscreen interocular delays required to null neural delays induced by differences in luminance between the eyes. Results are plotted as a function of the equivalent interocular difference in optical density. Error bars indicate 68% confidence intervals on each PSE (i.e. point of subjective equality) from 1000 bootstrapped datasets.

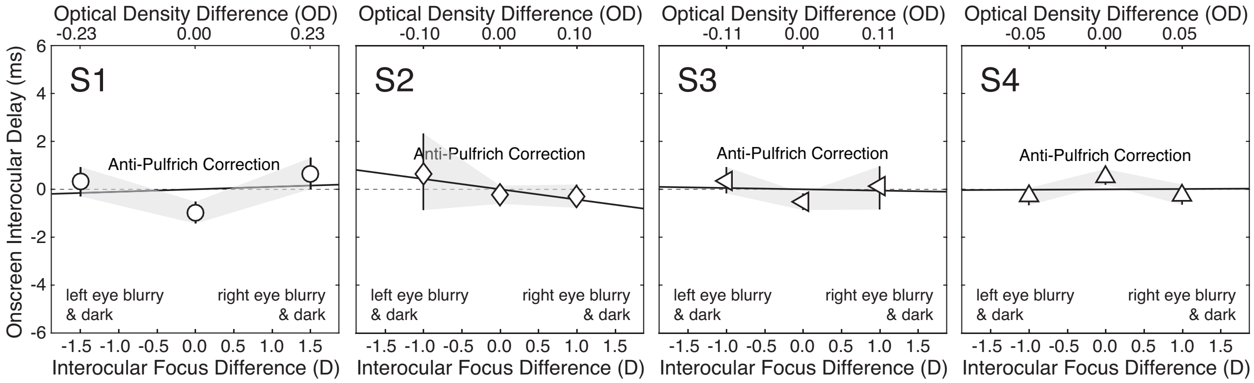

Figure S4. Anti-Pulfrich corrections with contact lenses eliminate the reverse Pulfrich effect for all four human observers (Exp. 4). Appropriately tinting the blurring lens eliminates the neural differences in processing speed caused by blur alone. Onscreen interocular delays are no longer required to null misperceptions of motion in depth. The anti-Pulfrich Each observer required a different anti-Pulfrich correction (i.e. a different optical density difference for each focus error difference) because the ratio of the regression slopes in the reverse and classic Pulfrich conditions (Exp. 1 & Exp. 3) differed for each observer (see Eq. 11). Error bars indicate 68% confidence intervals on each PSE (i.e. point of subjective equality) from 1000 bootstrapped datasets.

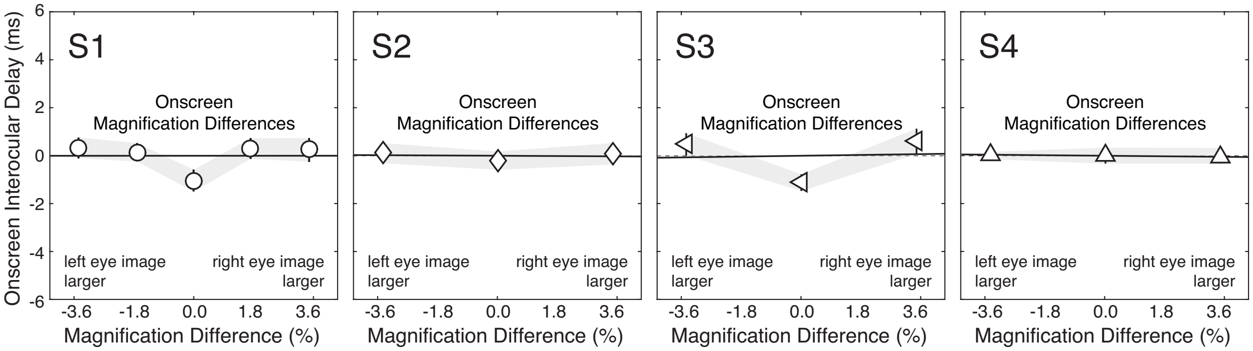

Figure S5. Magnification differences do not cause motion-in-depth misperceptions, for all four human observers (Exp. 5). Onscreen interocular delays equal zero for all interocular differences in magnification. Error bars indicate 68% confidence intervals on each PSE (i.e. point of subjective equality) from 1000 bootstrapped datasets.
